# Supplementary material for: DMRforPairs: identifying Differentially Methylated Regions between unique samples using array based methylation profiles
Source: BMC Bioinformatics. 2014 May 15;15:141. doi: 10.1186/1471-2105-15-141 (PMC4046028; doi:10.1186/1471-2105-15-141)
Supplement: Additional file 2 — DMRforPairs output for the comparison of A431-MCF7 and NA17018-NA17105. Please start from the HTML files in each folder. Available via the BMC Bioinformatics website. [file 1471-2105-15-141-S2.zip › 1394847754114233_MOESM2_ESM/A431_MCF7/figures/22380.pdf]

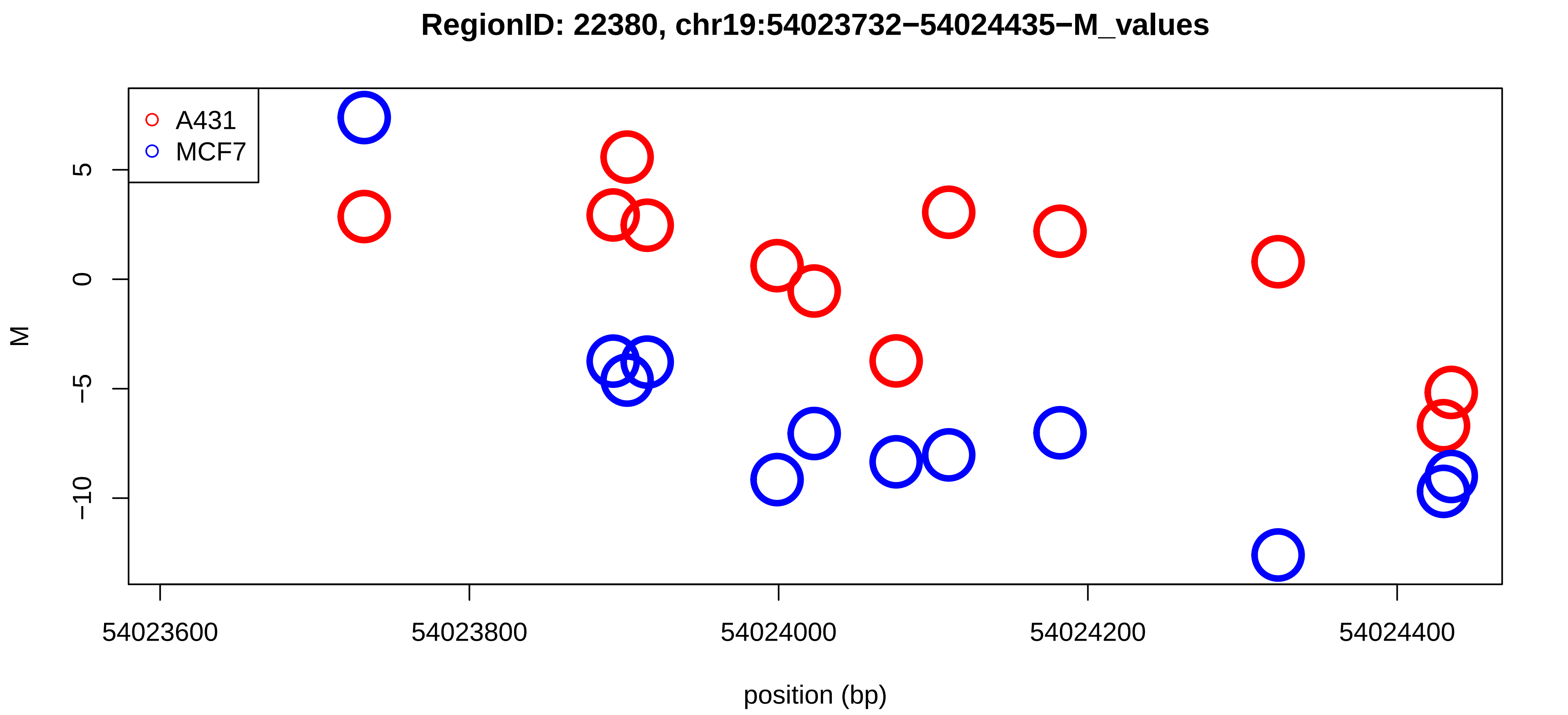

RegionID: 22380, chr19:54023732−54024435−M_values
A431
MCF7
M
5
0
−5
−10
54023600
54023800
54024000
54024200
54024400
position (bp)

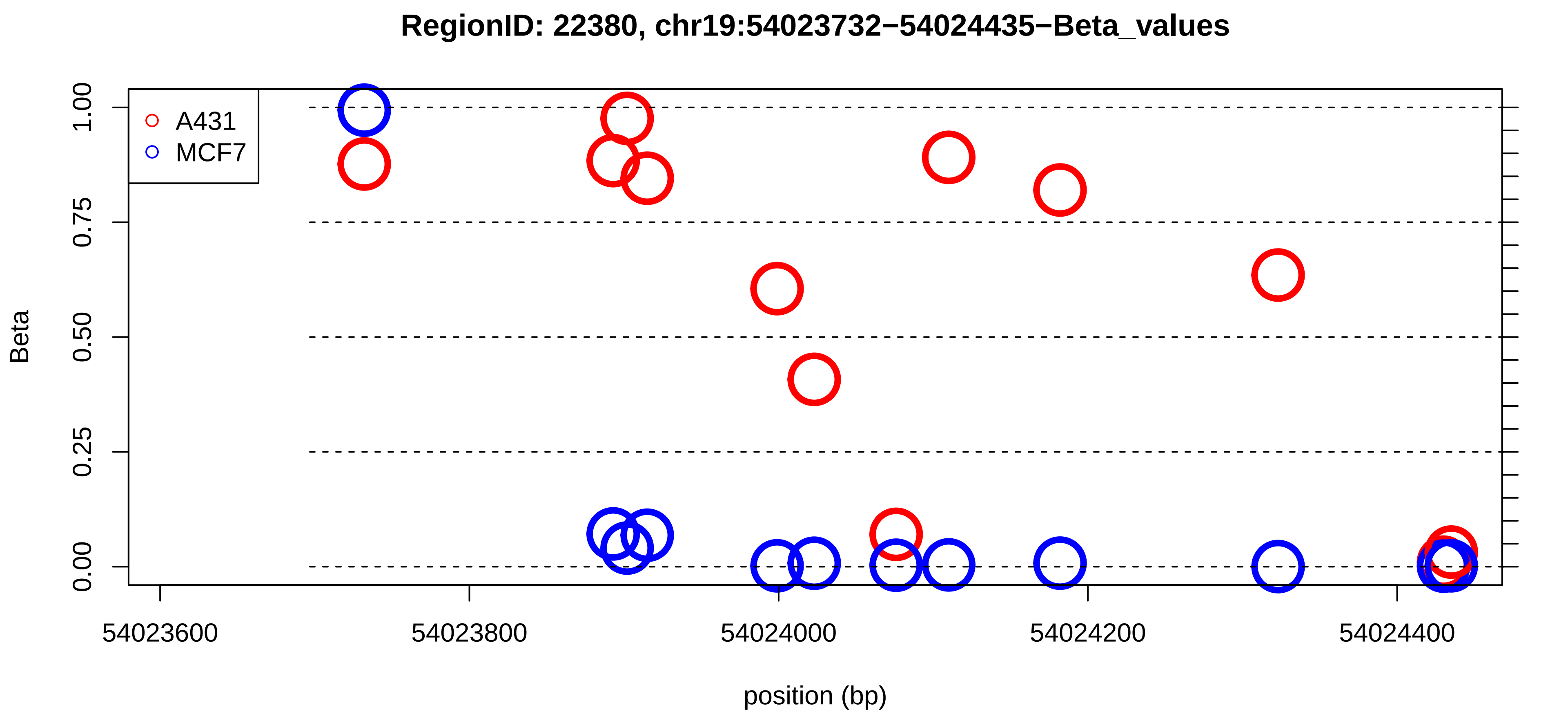

RegionID: 22380, chr19:54023732−54024435−Beta_values
A431
MCF7
Beta
1.00
0.75
0.50
0.25
0.00
54023600
54023800
54024000
54024200
54024400
position (bp)

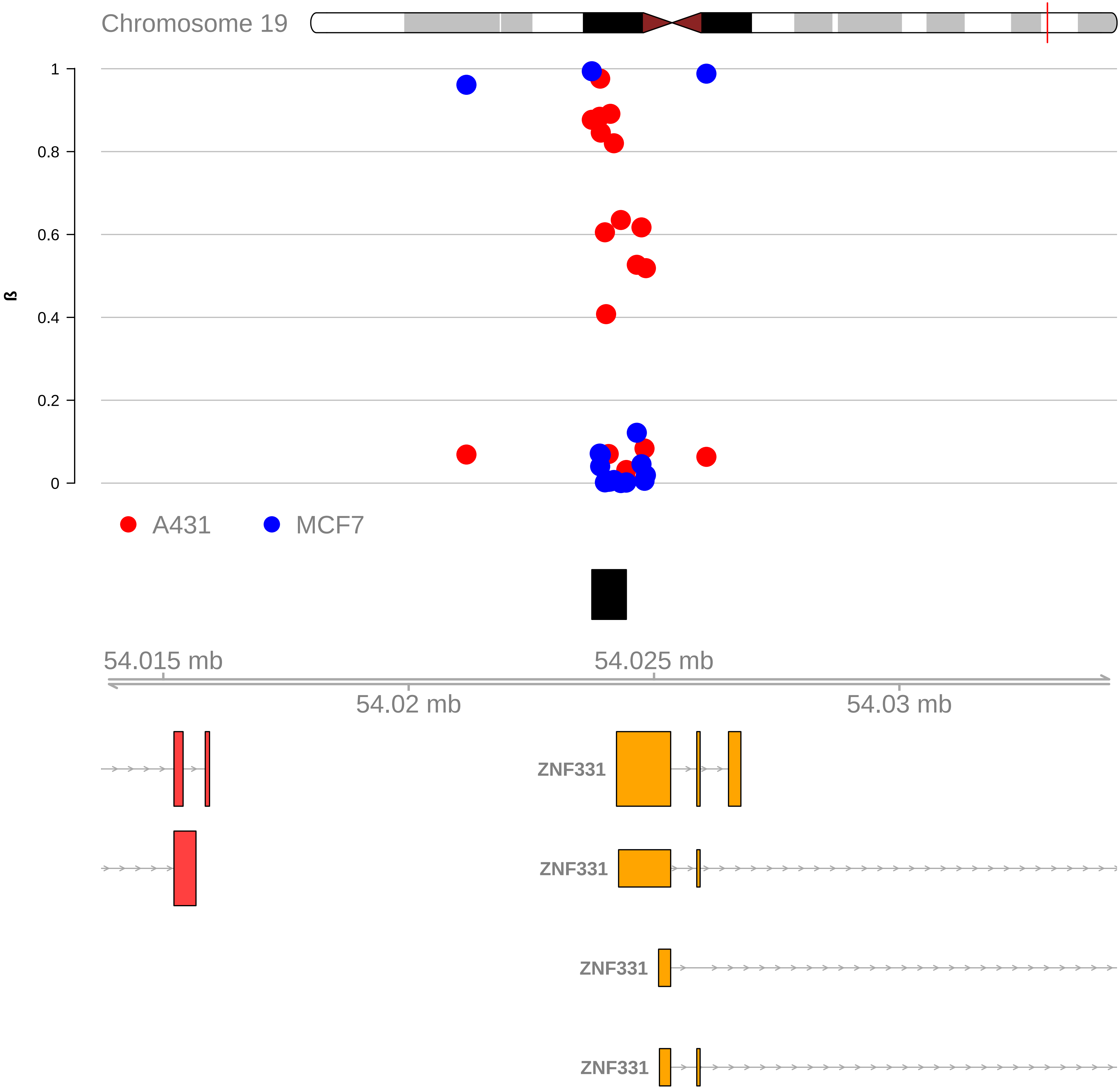

Chromosome 19
1
0.8
0.6
0.4
0.2
0
ß
A431
MCF7
54.015 mb
54.025 mb
54.02 mb
54.03 mb
ZNF331
ZNF331
ZNF331
ZNF331
